# Subtyping insomnia disorder with a population graph attention autoencoder: revealing two distinct biotypes

**DOI:** 10.3389/fnins.2026.1766155

**Published:** 2026-02-11

**Authors:** Heng Zhang, Hanbin Deng, Yiran Zhai, Jiang Zhang, Zixi Zhao, Liang Gong

**Affiliations:** 1 College of Electrical Engineering, Sichuan University, Chengdu, China; 2 Sichuan Institute of Computer Sciences, Chengdu, China; 3 College of Oxford Brookes, Chengdu University of Technology, Chengdu, China; 4 Department of Neurology, West China School of Medicine, Sichuan University Affiliated Chengdu Second People's Hospital, Sichuan University, Chengdu, Sichuan, China; 5 Sichuan Provincial Engineering Research Center of Brain-machine Interactive Neuromodulation, West China Hospital, Sichuan University, Chengdu, Sichuan, China

**Keywords:** graph attention autoencoder, gray matter volume, insomnia disorder, structural covariance network, structural MRI

## Abstract

Insomnia disorder (ID) is neurobiologically heterogeneous and often eludes characterization by traditional group-level neuroimaging. Subtyping based on neuroimaging and clinical data offers a promising strategy for identifying biologically and clinically meaningful ID subgroups. To address this need, we developed a Gray Matter Population Graph Attention Autoencoder (GM-PGAAE) to subtype insomnia disorder in a cohort comprising 140 patients diagnosed with ID and 57 matched healthy controls. Each subject was represented as a node defined by atlas-based gray matter (GM) volumes. Population edges combined imaging-derived intersubject correlations with clinical similarity via a Hadamard product, generating an adjacency matrix that jointly encodes structural and phenotypic relationships. A Graph Attention Autoencoder learned low-dimensional embeddings that adaptively weighted informative intersubject connections, and clustering these embeddings identified distinct subtypes. Regional and network-level differences were further assessed using Voxel-Based Morphometry (VBM) and individualized differential structural covariance networks (IDSCNs). Through this framework, two ID subtypes were identified. Compared with Subtype 2, Subtype 1 showed higher symptom severity and greater GM reductions–particularly in the cerebellar vermis, thalamus, middle occipital cortex, fusiform gyrus, and paracentral lobule–alongside negative associations between GM volume and clinical scores. IDSCNs further revealed reduced thalamocortical and subcortical Z-scores in Subtype 1, indicating subtype-specific network alterations. Overall, GM-PGAAE integrates structural MRI and clinical measures to derive individualized embeddings and delineate biologically distinct ID subtypes.

## 1 Introduction

Insomnia disorder (ID) is a prevalent sleep disorder characterized by difficulties in initiating or maintaining sleep, accompanied by significant daytime impairment (Morin and Benca, 2012). Despite its high prevalence, the neurobiological mechanisms underlying insomnia remain poorly understood, and clinical presentations are heterogeneous (Riemann et al., 2017). Traditional group-level analyses often fail to capture individual variability, limiting their utility for personalized diagnosis and intervention (Wager and Atlas, 2015). Data-driven subtyping approaches that integrate neuroimaging and clinical measures have the potential to identify biologically and clinically meaningful subgroups, thereby improving our understanding of insomnia heterogeneity (Van Someren, 2021; Blanken et al., 2019).

Previous efforts to characterize insomnia have primarily relied on either clinical assessments or conventional neuroimaging analyses. For instance, (Blanken et al. 2019) identified five insomnia subtypes using life-history and affective/personality traits, however, these subtypes were derived exclusively from clinical features and lacked neurobiological validation. Similarly, (Bjorøy et al. 2020) classified individuals with insomnia into symptom-based subtypes defined by difficulties with sleep onset, sleep maintenance, and early morning awakening, and reported subtype differences in anxiety, depression, and other clinical dimensions. However, this classification was also based solely on symptom profiles and lacked supporting structural or functional neuroimaging evidence.

Traditional voxel-wise or region-of-interest (ROI) analyses of structural MRI have identified gray matter (GM) alterations associated with insomnia, providing important insights into the neuroanatomical correlates of sleep disturbances. For example, (Altena et al. 2010) reported reduced GM in the orbitofrontal and parietal cortices in chronic insomnia patients, establishing a foundation for group-level voxel-based morphometry (VBM) studies. (Dai et al. 2018) compared GM changes following acute sleep deprivation and chronic insomnia, revealing both common and distinct alterations in the thalamus, cerebellum, and insula, yet without integrating multimodal data or network-level analyses. More recently, (Xu et al. 2024) combined VBM with resting-state functional connectivity to investigate chronic insomnia with comorbid anxiety, demonstrating GM changes in the cerebellum and insula alongside functional network abnormalities. In parallel, recent functional-connectome work further advances understanding of insomnia. A gradient-based study reported marked disruptions in large-scale functional gradients—particularly in the middle frontal gyrus, anterior cingulate cortex, nucleus accumbens, and occipito-temporal regions—and showed that these gradient features reliably predict sleep quality as well as anxiety and depression scores, underscoring their potential as clinical biomarkers (Wu et al., 2024). Despite these advances, most neuroimaging studies rely on group-level comparisons, which may overlook interindividual variability that is essential for identifying heterogeneity within insomnia populations.

However, both clinically driven and imaging-only approaches remain limited in their ability to capture the complex, multilevel heterogeneity of insomnia. Clinical subtyping lacks neurobiological support, whereas traditional imaging analyses focus on group-level differences and overlook individualized patterns of brain organization (Zhao et al., 2025; Bresser et al., 2025). These gaps highlight the need for analytical frameworks capable of jointly modeling subject-level clinical features and neuroanatomical relationships. Graph-based learning frameworks, particularly graph neural networks (GNNs), offer a powerful means to address limitations of traditional clinical and neuroimaging analyses (Velickovic et al., 2017; Scarselli et al., 2008). By modeling each subject as a node and defining edges based on intersubject similarity, population-graph approaches can jointly incorporate structural MRI features and clinical measures.

In this study, we sought to better characterize neurobiological heterogeneity in insomnia disorder by identifying clinically and structurally meaningful subtypes. We developed Gray Matter Population Graph Attention Autoencoder (GM-PGAAE) that integrates gray-matter (GM) features with key clinical assessments. The model learns low-dimensional embeddings that emphasize informative intersubject relationships, and clustering these embeddings reveals distinct insomnia subtypes. To assess the biological relevance of these subgroups, we further conducted voxel-based morphometry (VBM) analyses and constructed individualized differential structural covariance networks (IDSCNs) to examine subtype-specific regional and network-level alterations (Guo et al., 2023).

Using this framework, we identified two clinically and neurobiologically distinct insomnia subtypes. Compared with healthy controls (HC), Subtype 1 showed gray matter reductions primarily in limbic-temporal, insular, and cerebellar regions, predominantly in the left hemisphere. In contrast, Subtype 2 demonstrated a distinct pattern characterized by relative gray matter increases in occipital, cerebellar vermis, and thalamic systems, together with additional frontal midline involvement. Direct comparison between the two subtypes further revealed that Subtype 1 exhibited greater symptom severity and more pronounced gray matter reductions than Subtype 2 in regions implicated in emotion regulation, sensory processing, and arousal, including the cerebellar vermis, thalamus, middle occipital cortex, fusiform gyrus, and paracentral lobule. Gray matter volumes in these regions showed negative associations with clinical symptom scores, suggesting a structural basis for symptom burden. Moreover, IDSCN analyses revealed weakened thalamocortical and subcortical covariance in Subtype 1 relative to Subtype 2, indicating distinct network-level disruptions. Together, these convergent structural and network signatures provide biologically grounded evidence that insomnia disorder is not a unitary condition but comprises subgroups with potentially different underlying pathophysiological mechanisms.

Importantly, identifying insomnia subtypes carries direct clinical significance. These subgroups may help explain heterogeneous treatment responses, guide the development of more targeted behavioral or neurobiological interventions, and enable early recognition of individuals at risk for severe or persistent symptom trajectories. Our findings identify clinically meaningful and biologically validated insomnia subtypes, providing a foundation for precision medicine strategies in this disorder.

## 2 Methods

**Figure 1:**
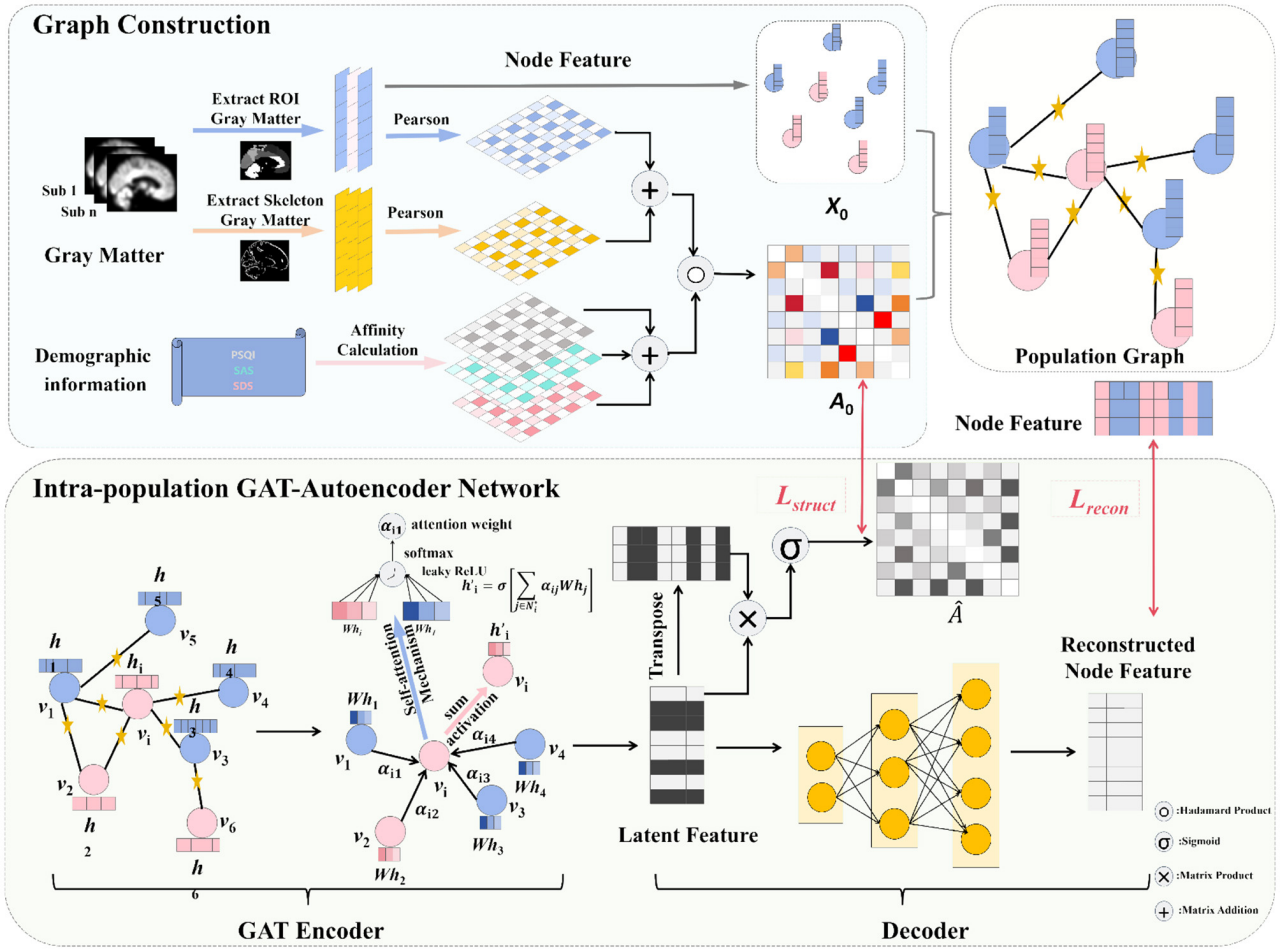
Overview of the proposed framework. Graph construction: each node represents the imaging information of an individual, and edges denote the inter-individual similarities based on imaging and clinical features. GAT-autoencoder network: the constructed population graph is then fed into a Graph Attention Network-based Autoencoder (GAT-AE), where the encoder adaptively aggregates information from neighboring nodes to obtain latent representations, and the decoder reconstructs the original node features. The learned latent representations are subsequently used for subtype identification.

We proposed GM-PGAAE for insomnia subtyping, as illustrated in Figure 1. This method integrates structural neuroimaging features and clinical information within a population graph learning framework. Each subject is represented as a node characterized by regional GM volumes derived from predefined regions of interest (e.g., AAL3 atlas) (Rolls et al., 2020). Because correlations based solely on ROI-level GM volumes may overlook subtle morphological patterns, we additionally incorporated skeletonized GM volumes, defining graph edges as the sum of the pairwise Pearson correlations computed from both feature types across subjects. To capture clinical similarity, the correlation matrix was further combined element-wise with scores from the Pittsburgh Sleep Quality Index (PSQI), Self-Rating Anxiety Scale (SAS), and Self-Rating Depression Scale (SDS) via a Hadamard product, yielding an adjacency matrix that jointly reflects neuroanatomical and psychometric relationships. A Graph Attention Autoencoder was then employed to learn low-dimensional latent embeddings from this population graph, where attention layers adaptively weight neighboring nodes to highlight the most informative intersubject connections. Finally, the latent representations were clustered to identify distinct insomnia subtypes, providing an interpretable and biologically grounded framework for data-driven characterization of insomnia heterogeneity.

To further validate the identified subtypes, voxel-wise VBM analyses were performed, and the relationships between subtypes and clinical characteristics were examined. In addition, IDSCNs were constructed to investigate group-level differences in interregional gray matter covariation patterns.

### 2.1 Participants and MRI data preprocessing

A total of 140 patients diagnosed with insomnia disorder and 57 matched healthy controls were included in this study.

All participants were recruited from the Neurology Outpatient Department of Chengdu Second People's Hospital (CSPH) and underwent a series of neuropsychological tests and MRI scans, consistent with our previous research (Zhong et al., 2025; Gong et al., 2025; Li et al., 2026). The study was approved by the Institutional Review Board Ethics Committee of Chengdu Second People's Hospital (ethics approval number: 2020021), and written informed consent was obtained from each subject. The demographic and clinical characteristics of the enrolled participants are summarized in Table 1.

**Table 1:** Demographic and clinical data of HC group and ID.

	**HC (*n* = 57)**	**ID (*n* = 140)**	***P*-value**
Age	34.75 ± 8.32 [21, 56]	36.69 ± 11.51 [18, 60]	0.2539^*a*^
Sex (F/M)	21/36	75/65	0.0485^*b*^
PSQI	3.56 ± 1.51 [0, 6]	13.19 ± 2.74 [8, 20]	< 0.0001^*a*^
SAS	38.12 ± 10.10 [25.0, 62.5]	49.25 ± 10.61 [26.0, 81.25]	< 0.0001^*a*^
SDS	33.72 ± 7.31 [25.0, 51.25]	50.38 ± 11.37 [25.0, 87.5]	< 0.0001^*a*^

Continuous variables are presented as mean ± standard deviation [range].

^*a*^: *P*-values were calculated using two-sample *t*-tests; ^*b*^: *P*-values were calculated using chi-square tests.

We used CAT12 for voxel-based morphological preprocessing of T1-weighted structural data (Gaser et al., 2024), which included the following steps: (1) skull stripping, denoising, and bias correction; (2) segmentation of T1-weighted images into gray matter, white matter, and cerebrospinal fluid; (3) registration of gray matter maps to the standard template using the diffeomorphic anatomical registration through exponentiated Lie algebra (DARTEL) algorithm, followed by resampling to a voxel size of 1.5 × 1.5 × 1.5 *mm*^3^; (4) estimation of total intracranial volume (TIV); and (5) spatial smoothing with an 8 mm full-width at half-maximum (FWHM) Gaussiankernel.

### 2.2 Population graph construction

We obtained gray matter and clinical information for *N* subjects. To specifically investigate heterogeneity within insomnia disorder, only patients diagnosed with insomnia disorder were included in the construction of the population graph (*N* = 140). Based on these data, a population graph *G* = (*X, A*) was constructed, where *X*∈ℝ^*N*×*F*^ represents the features of the *N* nodes (subjects), and *F* denotes the number of features per node. For each subject, gray matter was parcellated using the AAL3 atlas, and the gray matter volumes of 166 regions were extracted as features for each node, forming the original node feature matrix *X*_0_. During regional feature extraction, voxels were assigned to brain regions according to the atlas definition, and each voxel contributed to a single region, thereby preventing overlapping regional contributions.

*A*∈ℝ^*N*×*N*^ denotes the edges between different nodes. To construct the edge weight adjacency matrix, we first computed an imaging feature similarity matrix by combining two components: (1) the Pearson correlation of gray matter volumes across ROIs defined by the AAL3 atlas, and (2) the Pearson correlation of gray matter volumes along the cortical skeleton. The cortical skeleton was derived using a Tract-Based Spatial Statistics-style (TBSS-style) skeletonization procedure. A mean GM volume map was first generated across all subjects, and a cortical skeleton was subsequently extracted using the tbss_skeleton function in FSL. This procedure projects individual GM volume values onto the center of the cortical mantle, yielding a skeletonized GM representation that captures core cortical structures with maximal inter-subject correspondence. The resulting skeleton mask was then applied to each subject to extract subject-specific skeletonized GM volumes for subsequent analyses. The sum of these correlations forms the imaging similarity matrix, denoted as *S*_*I*_, which was designed to preserve complementary structural information captured at both regional (ROI-based GM volume) and cortical-core (skeletonized GM) levels, while avoiding premature feature-level fusion that may obscure their distinct contributions.

For clinical information, a clinical similarity matrix *C*_*d*_ was constructed to represent the strength of the relationship between subjects. Specifically, for each clinical measure *d*∈*D*, where *D* = {PSQI, SAS, SDS}, subjects were considered similar if the absolute difference in scores was below a threshold. An adaptive approach was used to determine the threshold for each measure, in which the 25th percentile of score differences was set as the similarity threshold, a strategy commonly adopted in population graph construction to suppress weak or noisy inter-subject relationships while preserving the most comparable subject pairs (Said et al., 2023; Tsai, 2018). Binary similarity matrices were constructed for each measure and combined to obtain the final clinical similarity matrix *C*_*d*_. The imaging similarity matrix *S*_*I*_ and clinical similarity matrix *C*_*d*_ were then combined using the Hadamard product to obtain the final adjacency matrix:


$$\begin{array}{l} A=S_{I}⊙C_{d} \end{array}$$ (1)

where ⊙ denotes the element-wise (Hadamard) product. To enhance sparsity, only the top 20% strongest connections were retained to form the final adjacency matrix *A*_0_. Percentile-based sparsification is widely used in brain network analysis and population graph modeling to improve graph stability and suppress spurious inter-subject correlations (Li et al., 2025), thereby yielding a population graph that integrates both imaging and clinical information.

### 2.3 Graph attention autoencoder

#### 2.3.1 GAT-based encoder

The encoder leverages a Graph Attention Network (GAT) to learn node embeddings by adaptively aggregating information from neighboring subjects defined by the population graph. The constructed graph provides a sparse binary connectivity structure, indicating candidate inter-subject relationships for message passing. Unlike standard graph convolutional models that aggregate neighbor information with fixed or uniform weights, GAT employs an attention mechanism to assign data-driven importance to different neighboring nodes during feature aggregation. This design is particularly suitable for population graphs derived from imaging and clinical similarity, where graph connectivity reflects potential relationships but their relative importance is not known a priori. By learning adaptive attention weights, the model can emphasize more informative inter-subject connections and better capture individual-level heterogeneity for subtype identification. As shown in Figure 1, given an input feature matrix *X*∈ℝ^*N*×*F*^, the representation of node *v*_*i*_ is updated through the attention-based aggregation mechanism as follows:


$$\begin{array}{l} h_{i}^{\prime }={∥}_{k=1}^{K}\sigma \left(\underset{j\in \mathbb{N}i^{*}}{\sum }\alpha_{ij}^{\left(k\right)}W^{\left(k\right)}h_{j}\right) \end{array}$$ (2)

where $W^{\left(k\right)}\in {\mathbb{R}}^{d_{s}\times F}$ denotes the learnable weight matrix of the *k*-th attention head, σ(·) represents a nonlinear activation function (ELU in our implementation), and ∥ denotes the concatenation operation across *K* independent attention heads. The normalized attention coefficient $\alpha_{ij}^{\left(k\right)}$ quantifies the importance of node *v*_*j*_ to node *v*_*i*_ for the *k*-th head, computed as:


$$\begin{array}{l} e_{ij}^{\left(k\right)}=\text{LeakyReLU}\left(a^{\left(k\right)⊤}\left[W^{\left(k\right)}h_{i}∥W^{\left(k\right)}h_{j}\right]\right) \end{array}$$ (3)

$$\begin{array}{l} \alpha_{ij}^{\left(k\right)}=\frac{\exp\left(e_{ij}^{\left(k\right)}\right)}{\sum_{m\in \mathbb{N}i^{*}}\exp\left(e{im}^{\left(k\right)}\right)} \end{array}$$ (4)

where $a^{\left(k\right)}\in {\mathbb{R}}^{2d_{s}}$ is a learnable attention vector of the *k*-th head. The LeakyReLU function introduces nonlinearity into the attention mechanism, while the softmax operation ensures normalization over all neighbors of node *v*_*i*_. To capture higher-order neighborhood dependencies, multiple GAT layers are stacked in the encoder, where each layer refines node embeddings based on the aggregated representations from the previous layer. After *L* layers of GAT learning, the final node representation $h_{i}^{\left(L\right)}$ is obtained. The latent embedding *z*_*i*_ for node *v*_*i*_ is derived through a fully connected transformation:


$$\begin{array}{l} z_{i}=W_{\mu }h_{i}^{\left(L\right)}+b_{\mu }, \end{array}$$ (5)

where *W*_μ_ and *b*_μ_ are learnable parameters.

#### 2.3.2 Decoder

The learned latent representation *z*_*i*_ is then fed into a decoder for feature reconstruction. The decoder aims to recover the original node features from the low-dimensional latent embeddings while preserving the structural information encoded by the encoder. It is implemented as a multi-layer neural network composed of several fully connected layers followed by layer normalization and nonlinear activation functions. Given the latent representation $z_{i}\in {\mathbb{R}}^{d_{z}}$, the reconstructed feature ${\hat{x}}_{i}$ is computed as:


$$\begin{array}{l} {\hat{x}}_{i}=f_{\text{Decoder}}\left(z_{i}\right), \end{array}$$ (6)

where *f*_Decoder_(·) denotes the nonlinear mapping learned by the decoder network.

#### 2.3.3 Loss Function

To train the proposed GAT-based autoencoder, two loss terms are jointly optimized: a feature reconstruction loss and a structure reconstruction loss.

The feature reconstruction loss ensures that the decoder can accurately recover the original node features from their latent embeddings. It is defined as the mean squared error (MSE) between the reconstructed features $\hat{X}$ and the input features *X*_0_:


$$\begin{array}{l} L_{\text{recon}}=\frac{1}{N}\overset{N}{\underset{i=1}{\sum }}|{\hat{x}}_{i}-x_{i}{|}^{2} \end{array}$$ (7)

where *N* is the number of nodes, *x*_*i*_ and ${\hat{x}}_{i}$ denote the original and reconstructed features of node *v*_*i*_, respectively.

To preserve the graph's topological structure in the latent space, a structure reconstruction loss is introduced. The adjacency matrix $A_{0}\in {\mathbb{R}}^{N\times N}$ is reconstructed from the latent representations $Z={\left[z_{1},z_{2},\ldots ,z_{N}\right]}^{⊤}$ using an inner-product decoder:


$$\begin{array}{l} Â=\sigma \left(ZZ^{⊤}\right), \end{array}$$ (8)

where σ(·) denotes the sigmoid activation function. The structure reconstruction loss is then formulated as the binary cross-entropy (BCE) between the predicted and true adjacency matrices:


$$\begin{array}{l} L_{\text{struct}}=-\frac{1}{N^{2}}\overset{N}{\underset{i=1}{\sum }}\overset{N}{\underset{j=1}{\sum }}\left[A_{ij}\log\left({Â}_{ij}\right)+\left(1-A_{ij}\right)\log\left(1-{Â}_{ij}\right)\right] \end{array}$$ (9)

The overall loss function combines these two objectives with a balancing coefficient λ:


$$\begin{array}{l} L_{\text{total}}=L_{\text{recon}}+\text{λ}L_{\text{struct}}, \end{array}$$ (10)

where controls the contribution of structural preservation during training.

#### 2.3.4 Model architecture and implementation details

The proposed GM-PGAAE framework was implemented as a graph attention autoencoder consisting of an encoder and a decoder. The encoder comprised four stacked GAT layers with hidden dimensions of 256, 128, 64, and 32, respectively. The numbers of attention heads in the four layers were set to 4, 4, 1, and 1. Each GAT layer was followed by a layer normalization operation and an ELU activation function, and no dropout was applied during training. The output of the final GAT layer was projected into a latent space of dimension 16 via a fully connected layer, yielding subject-specific latent representations. The decoder was implemented as a multilayer perceptron with three hidden layers of sizes 32, 64, and 128, followed by a linear output layer that reconstructed the original node features. Layer normalization and leaky ReLU activations were applied between decoder layers. All models were trained using the Adam optimizer with a learning rate of 0.001. The training objective combined a node feature reconstruction loss and a graph structure reconstruction loss. The two loss terms were combined using a structural loss coefficient λ, which was selected via post hoc model selection based on the silhouette coefficient of the resulting latent representations.

### 2.4 Subtype identification and statistical analysis

The latent representations $Z={\left\{z_{i}\right\}}_{i=1}^{N}$, where each $z_{i}\in {\mathbb{R}}^{d_{z}}$, were obtained from the trained GAT Autoencoder and subsequently used as input features for subtype identification via K-means clustering. In this study, the latent dimension was empirically set to *d*_*z*_ = 16, which provided a compact yet informative representation of individual structural characteristics. Two hyperparameters were considered to influence the clustering results: the structural loss weight λ used during GAT Autoencoder training, and the predefined number of clusters *C*. Specifically, λ was varied from 0.0 to 1.0, and *C* was set from 2 to 5. The optimal clustering solution was determined by selecting the combination that achieved the highest silhouette coefficient, indicating the most compact and well-separated cluster structure. Notably, the silhouette coefficient was used solely for post hoc evaluation and model selection, and was not involved in network training or loss optimization.

After defining the ID subtypes, voxel-wise two-sample *t*-tests were first performed between Subtype 1 and Subtype 2 to identify subtype-specific structural differences, while controlling for age, sex, and total intracranial volume (TIV). In addition, each ID subtype was separately compared with healthy controls using voxel-wise two-sample *t*-tests with the same covariates, in order to characterize subtype-specific abnormal structural patterns. All voxel-wise results were thresholded at *p* < 0.05 with Bonferroni correction and a minimum cluster size greater than 50 voxels. Further analyses examined gray matter volume differences among subtypes in the significant brain regions, as well as between-group differences in clinical information. In addition, correlation analyses were performed between gray matter volumes in subtype-differentiated regions and clinical scale scores to explore potential associations between structural alterations and clinical characteristics.

### 2.5 Subsampling-based stability analysis

To assess the robustness of the learned embeddings and the stability of subtype assignments, we performed a subsampling-based stability analysis. Specifically, in each run, a random subset of subjects (80% of the ID subjects) was selected without replacement, and the GAT autoencoder was retrained using the corresponding induced subgraph, with all model hyperparameters (including network architecture and the structural loss weight λ) fixed to the optimal configuration identified in the main analysis. This procedure was repeated for 30 independent subsampling runs. Latent representations obtained from each run were then clustered using K-means with the number of clusters fixed to the optimal value determined in the main analysis. Clustering stability was evaluated by computing the adjusted Rand index (ARI) between all pairs of subsampling runs. The ARI quantifies the agreement between two clustering solutions while correcting for chance, with values close to 1 indicating highly consistent cluster assignments and values near 0 indicating agreement no better than random. For each pair of runs, the ARI was calculated only on the overlapping subjects that appeared in both runs, thereby measuring the consistency of subtype assignments under data perturbation. In total, this resulted in 435 pairwise comparisons across subsampling runs. The distribution of pairwise ARI values was used as an indicator of overall clustering stability. In addition, to examine whether stability depended on the number of overlapping subjects, we analyzed the relationship between ARI values and the corresponding overlap size across subsampling runs.

### 2.6 Subject-specific individualized differential structural covariance network construction

**Figure 2:**
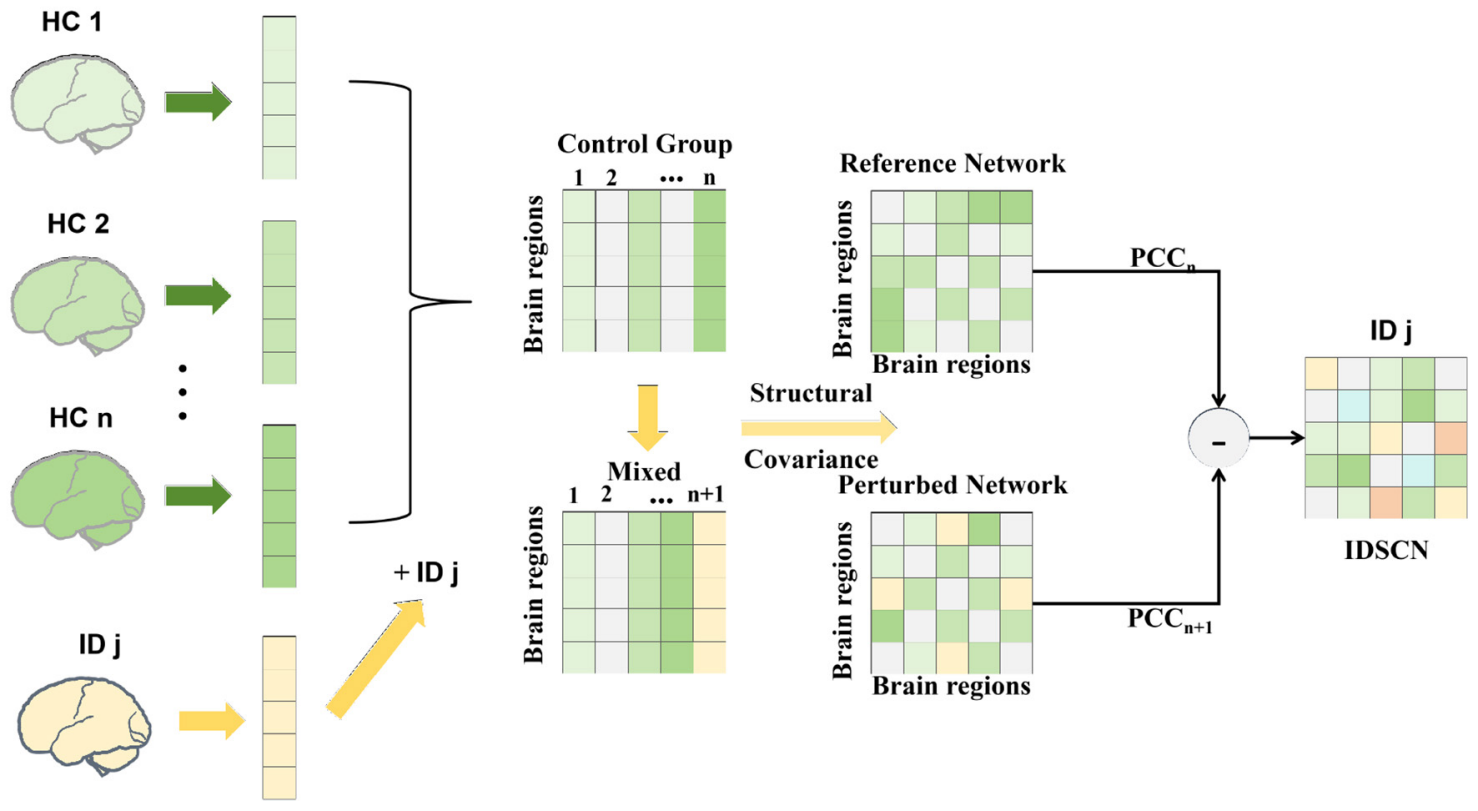
Individual differential structural covariance networks (IDSCNs) were constructed to reveal subtype-specific alterations in gray matter covariance patterns.

To investigate subject-specific alterations in structural covariance patterns between the ID and HC groups, we constructed individualized differential structural covariance networks following a data-driven framework adapted from a recently proposed bioinformatics approach (Liu et al., 2021). The overall framework of the IDSCN construction process is illustrated in Figure 2.

First, the entire brain was parcellated into 166 ROIs using the AAL3 atlas. Based on the HC group (*n* subjects), a reference structural covariance network was generated by calculating the partial Pearson's correlation coefficients of GMV across all ROI pairs while controlling for age, sex, and TIV. This yielded a 166 × 166 reference matrix, denoted as *PCC*_*n*_. For each ID participant *j*, a perturbed network *PCC*_*n*+1_ was then constructed by adding that subject to the HC group (i.e., *n* HCs + 1 ID). The individual-level contribution to the network was captured by the difference between the perturbed and reference networks:


$$\begin{array}{l} \text{Δ}PCC_{n}=PCC_{n+1}-PCC_{n}. \end{array}$$ (11)

Theoretically, Δ*PCC*_*n*_ follows a symmetric “volcano” distribution with Gaussian-like tails, allowing for statistical inference. For each edge, a *Z*-score was computed as:


$$\begin{array}{l} Z=\frac{\text{Δ}PCC_{n}}{\frac{1-PCC_{n}^{2}}{n-1}}, \end{array}$$ (12)

where *n* denotes the number of HC subjects. In the resulting IDSCN of participant *j*, each edge weight corresponded to the *Z*-score from Equation 12. The associated *p*-value for each edge was derived from the *Z*-distribution, and statistically significant deviations from the reference network were identified at *p* < 0.05, corrected for multiple comparisons using the FDR procedure. This process yielded an individualized IDSCN for every ID subject.

After obtaining all IDSCNs, we quantified the number of significantly altered edges (*p* < 0.05, Bonferroni corrected) per participant. To identify common network alterations across the ID group, we retained the top 20 edges that were significantly changed in at least 33 individuals for subsequent analyses, following the approach described in (Liu et al., 2021). Between-subtype differences in these edges were assessed using two-sample *t*-tests, and subtype-specific connections were further examined using Pearson correlation analyses with clinical measures within the ID group. Statistical significance for correlation analyses was set at *p* < 0.05, corrected by the FDR method. As an additional exploratory analysis, we also examined whether IDSCN features could be used directly for subtype identification. Specifically, Z-scores of the top 20 most altered structural covariance edges were extracted for each subject and used as input features for K-means clustering, with the number of clusters fixed to two for consistency with the main analysis.

## 3 Results

### 3.1 Distinct subtypes in insomnia disorder

**Figure 3:**
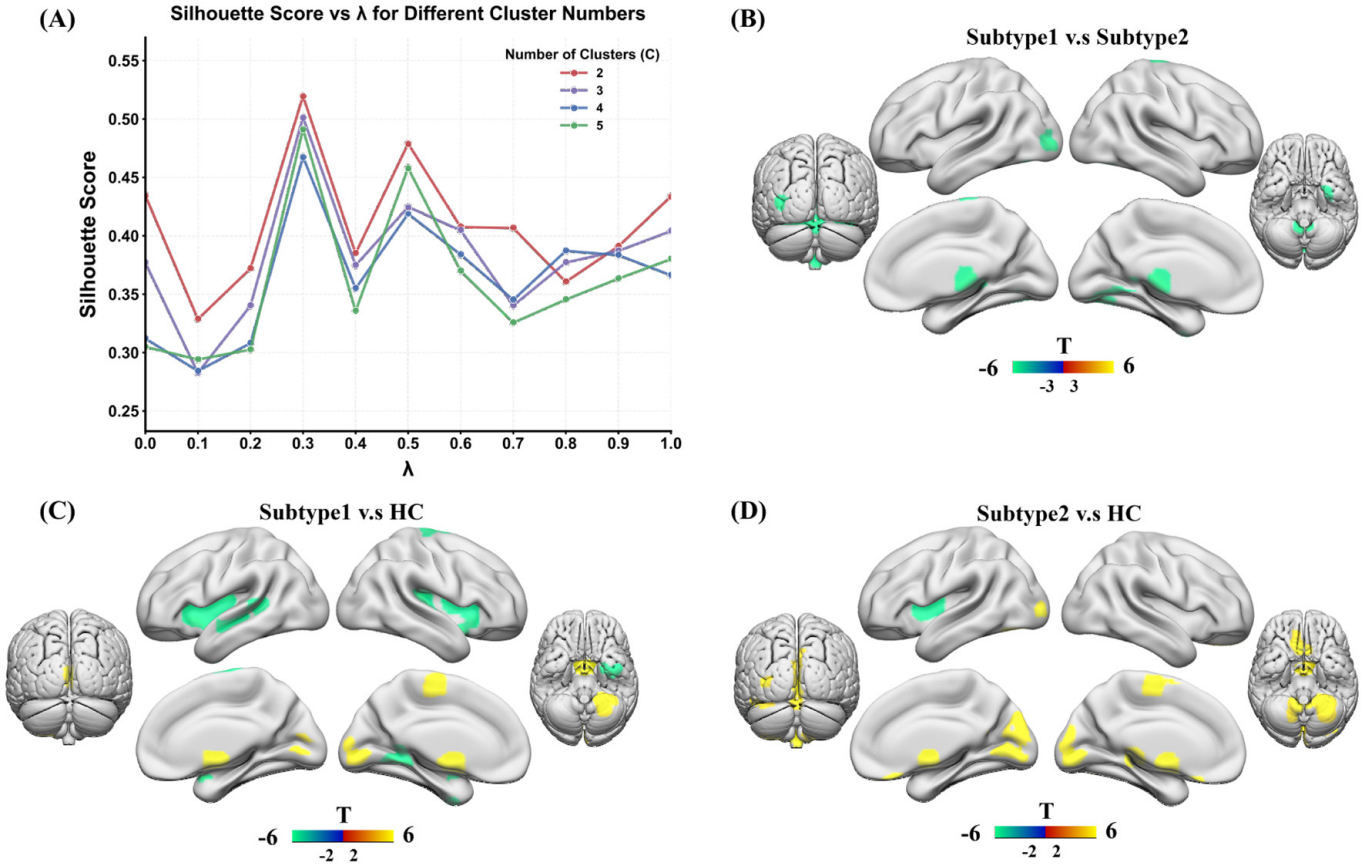
Overview of subtyping and GMV alterations in ID. **(A)** Visualization of the optimal hyperparameters, including the structural loss weight (λ) used during GAT Autoencoder training and the predefined number of clusters (*C*) for *K*-means clustering. Using these optimal parameters, two ID subtypes were identified: Subtype 1 (*n* = 90) and Subtype 2 (*n* = 50). **(B)** Voxel-wise statistical comparisons between the two subtypes revealed that Subtype 1 exhibited reduced gray matter volume relative to Subtype 2 in the *Vermis 6*, right *medial pulvinar nucleus of the thalamus (Thal.PuM.R)*, left *middle occipital gyrus (Occipital.Mid.L)*, left *fusiform gyrus (Fusiform.L)*, right *Cerebellum 9*, and right *paracentral lobule (Paracentral.Lobule.R)*. **(C)** Compared with HC, Subtype 1 showed significant gray matter reductions in the *left insula, putamen, superior temporal gyrus, lingual gyrus*, and *fusiform gyrus*, predominantly in the left hemisphere, as well as in cerebellar regions including *Cerebellum 4-5* and *Cerebellum 8*. **(D)** Subtype 2 demonstrated widespread gray matter increases in the *occipital cortex* (including the *calcarine, lingual*, and *cuneus* regions), cerebellar vermis areas (*Vermis 4-5, Vermis 6*, and *Vermis 7*), and thalamic subregions (*Thal.PuM.L, Thal.PuI.L*, and *Thal.PuA.L*). Additionally, gray matter alterations were observed in the *rectus gyrus* and *supplementary motor area*.

As illustrated in Figure 3A, two hyperparameters were evaluated to determine the optimal clustering configuration: the structural loss weight λ used during the GAT Autoencoder training and the predefined number of clusters *C* in the K-means clustering. The silhouette coefficient was calculated under different parameter combinations, and the optimal setting was obtained at λ = 0.3 and *C* = 2, where the clustering achieved the highest silhouette score, indicating the best separation and compactness between clusters. Based on this optimal configuration, the learned latent representations were subjected to K-means clustering. The silhouette coefficient was used to guide the selection of the clustering solution, resulting in two distinct insomnia subtypes:Subtype 1 (*n* = 90, 57 males; mean age = 38.03 ± 11.69 years) and Subtype 2 (*n* = 50, 18 males; mean age = 34.26 ± 10.76 years). Significant differences were observed between the two subtypes in terms of sex (*p* = 0.003), PSQI (*p* = 0.0004), SAS (*p* < 0.0001), and SDS (*p* < 0.0001), whereas age showed a trend toward difference but did not reach statistical significance (*p* = 0.064) (Table 2).

**Table 2:** Demographic and clinical data of Subtype1 and Subtype2.

	**Subtype1 (*n* = 90)**	**Subtype2 (*n* = 50)**	***P*-value**
Age	38.03 ± 11.69 [18, 60]	34.26 ± 10.76 [20, 60]	0.0638^*a*^
Sex (F/M)	33/57	32/18	0.0034^*b*^
PSQI	13.80 ± 2.32 [8, 19]	12.10 ± 3.07 [8, 20]	0.0004^*a*^
SAS	53.18 ± 7.07 [35.0, 81.25]	42.16 ± 12.13 [26.0, 70.0]	< 0.0001^*a*^
SDS	53.72 ± 8.22 [36.0, 87.5]	44.37 ± 13.57 [25.0, 72.5]	< 0.0001^*a*^

Continuous variables are presented as mean ± standard deviation [range].

^*a*^: *P*-values were calculated using two-sample *t*-tests; ^*b*^: *P*-values were calculated using chi-square tests.

**Figure 4:**
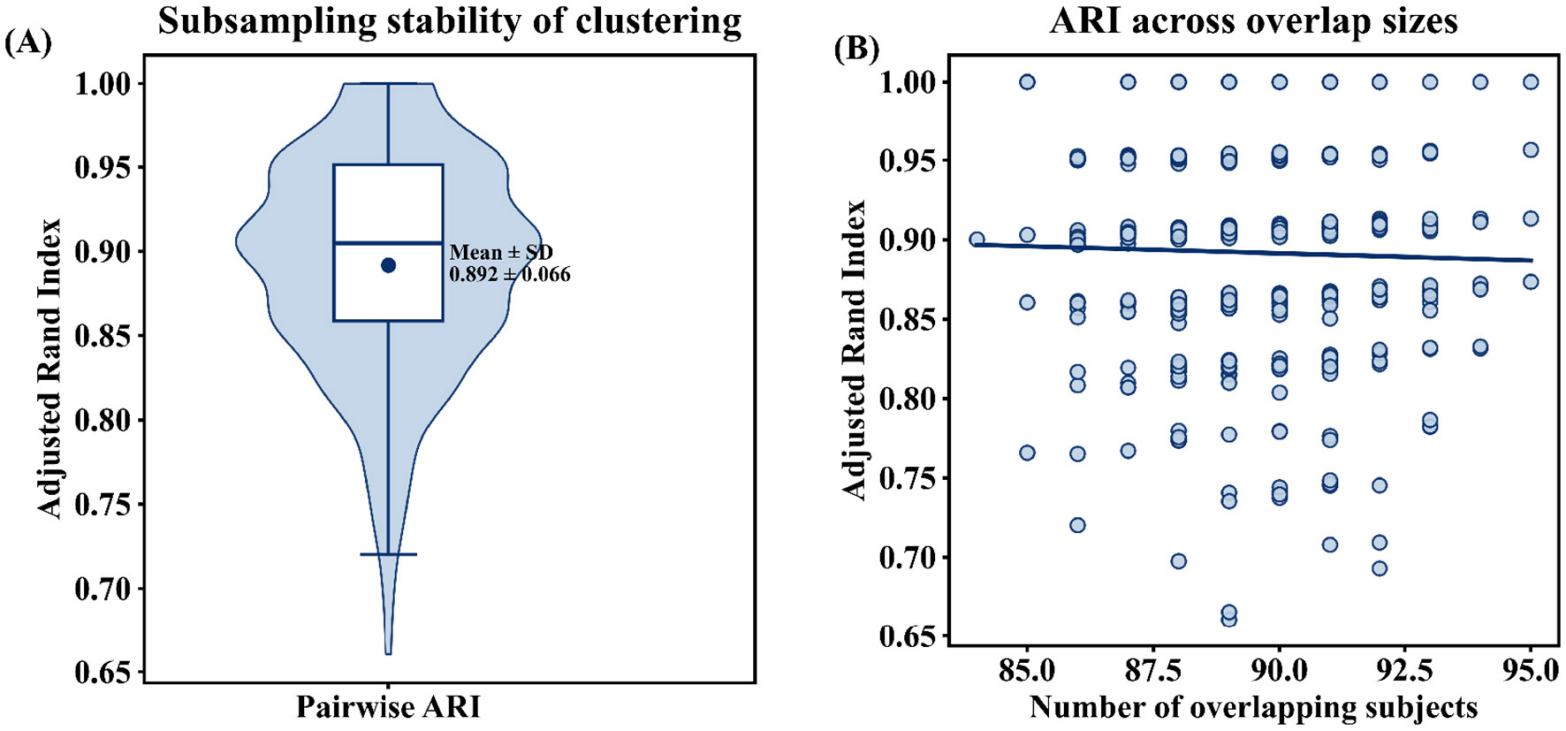
Subsampling-based stability analysis of insomnia subtype clustering. **(A)** Distribution of pairwise adjusted Rand index (ARI) values across all pairs of subsampling runs, illustrating the overall stability of subtype assignments under data perturbation. The central box indicates the interquartile range, the horizontal line denotes the median, and the dot represents the mean ARI. **(B)** Relationship between ARI values and the number of overlapping subjects between subsampling runs. Each point represents one pairwise comparison, and the fitted regression line indicates that clustering stability remains largely consistent across different overlap sizes.

To assess the robustness of the identified subtypes, we conducted a subsampling-based stability analysis. Pairwise comparisons across subsampling runs showed consistently high adjusted Rand index (ARI) values (mean ≈0.89), indicating stable subtype assignments under data perturbation (Figure 4A). In addition, ARI values were not strongly dependent on the number of overlapping subjects between runs (Figure 4B), supporting the robustness of the clustering results.

Furthermore, analysis of the learned attention coefficients revealed systematic differences between intra- and inter-subtype connections. As shown in Supplementary Figure S2, attention weights associated with inter-subtype edges were significantly higher than those within subtypes, as confirmed by a permutation-based statistical test (*p* < 0.001). This pattern indicates that, under the binary population-graph topology, attention coefficients are learned in a data-driven manner to adaptively reweight inter-subject information during representation learning, rather than trivially reinforcing within-subtype similarity.

Figure 3B presents the voxel-wise statistical comparison between the two identified subtypes, highlighting distinct gray matter regions that significantly differed between them. Specifically, Subtype 1 exhibited reduced gray matter volume compared with Subtype 2 in the *Vermis 6*, right *medial pulvinar nucleus of the thalamus (Thal.PuM.R)*, left *middle occipital gyrus (Occipital.Mid.L)*, left *fusiform gyrus (Fusiform.L)*, right *Cerebellum 9*, and right *paracentral lobule (Paracentral.Lobule.R)*. Furthermore, Figures 3C, D depict the voxel-level group differences between each subtype and HC. Compared with HC, Subtype 1 showed significant gray matter reductions in the *left insula, putamen, temporal superior gyrus, lingual gyrus*, and *fusiform gyrus*, predominantly in the left hemisphere, along with alterations in cerebellar regions such as *Cerebellum 4_5* and *Cerebellum 8*. In contrast, Subtype 2 demonstrated extensive gray matter increases in the *occipital cortex* (including the *calcarine, lingual*, and *cuneus* regions), as well as in cerebellar vermis areas (*Vermis 4_5, Vermis 6*, and *Vermis 7*), and thalamic subregions (*Thal.PuM.L, Thal.PuI.L*, and *Thal.PuA.L*). Additionally, gray matter differences were also observed in the *rectus gyrus* and *supplementary motor area*.

Together, these results indicate that the two insomnia subtypes are characterized by distinct and non-overlapping structural alteration patterns. Subtype 1 primarily exhibits gray matter atrophy in limbic-temporal and subcortical circuits, whereas Subtype 2 shows alterations centered in occipital-cerebellar and thalamic regions, suggesting distinct neuroanatomical mechanisms underlying insomnia disorder.

### 3.2 Comparison with baseline methods

To contextualize the effectiveness of the proposed GM-PGAAE framework, we compared it with several baseline approaches, including alternative graph neural network autoencoders (GCN-AE; Kipf, 2016 and GraphSAGE-AE; Hamilton et al., 2017) and non-graph clustering methods based on ROI gray matter volume features (K-means and PCA(16)+K-means (Abdi and Williams, 2010). All methods were evaluated under the same clustering configuration (*C* = 2). As summarized in Table 3, GM-PGAAE achieved the highest silhouette coefficient (0.520), indicating improved cluster compactness and separation relative to both graph-based and non-graph baselines. In addition, GM-PGAAE showed high clustering stability under subsampling perturbations (ARI = 0.892 ± 0.066), comparable to or exceeding that of the alternative methods. At the clinical level, graph-based approaches in general yielded subtype solutions with significant differences on at least one symptom scale, supporting the utility of population graph modeling for capturing clinically relevant heterogeneity. Notably, GM-PGAAE-derived subtypes demonstrated consistent differentiation across all three core clinical measures (PSQI, SAS, and SDS), together with favorable clustering compactness and stability. These results suggest that attention-based population graph representations may provide a more coherent and robust basis for subtype discovery in insomnia disorder.

**Table 3:** Comparison of GM-PGAAE with baseline methods for subtype discovery in insomnia disorder.

**Method**	**Representation**	***C***	**Silhouette**	**Stability (ARI)**	**PSQI**	**SAS**	**SDS**	**Duration**
**GAT-AE**	Population graph latent	2	**0.520**	**0.892** **±0.066**	**✓**	**✓**	**✓**	×
GCN-AE	Graph latent	2	0.347	0.774 ± 0.300	✓	✓	✓	×
GraphSAGE-AE	Graph latent	2	0.265	0.886 ± 0.028	×	✓	✓	×
PCA(16)+KMeans	PCA(ROI GMV)	2	0.326	0.835 ± 0.058	×	×	×	×
KMeans (ROI GMV)	ROI GMV (166D)	2	0.290	0.833 ± 0.060	×	×	×	×

Silhouette coefficient reflects cluster compactness and separation.

Stability was assessed using subsampling-based pairwise adjusted Rand index (ARI), reported as mean ± standard deviation.

✓ indicates a significant subtype difference after FDR correction; × indicates non-significance.

### 3.3 Distinct GMV and clinical characteristics between subtypes

**Figure 5:**
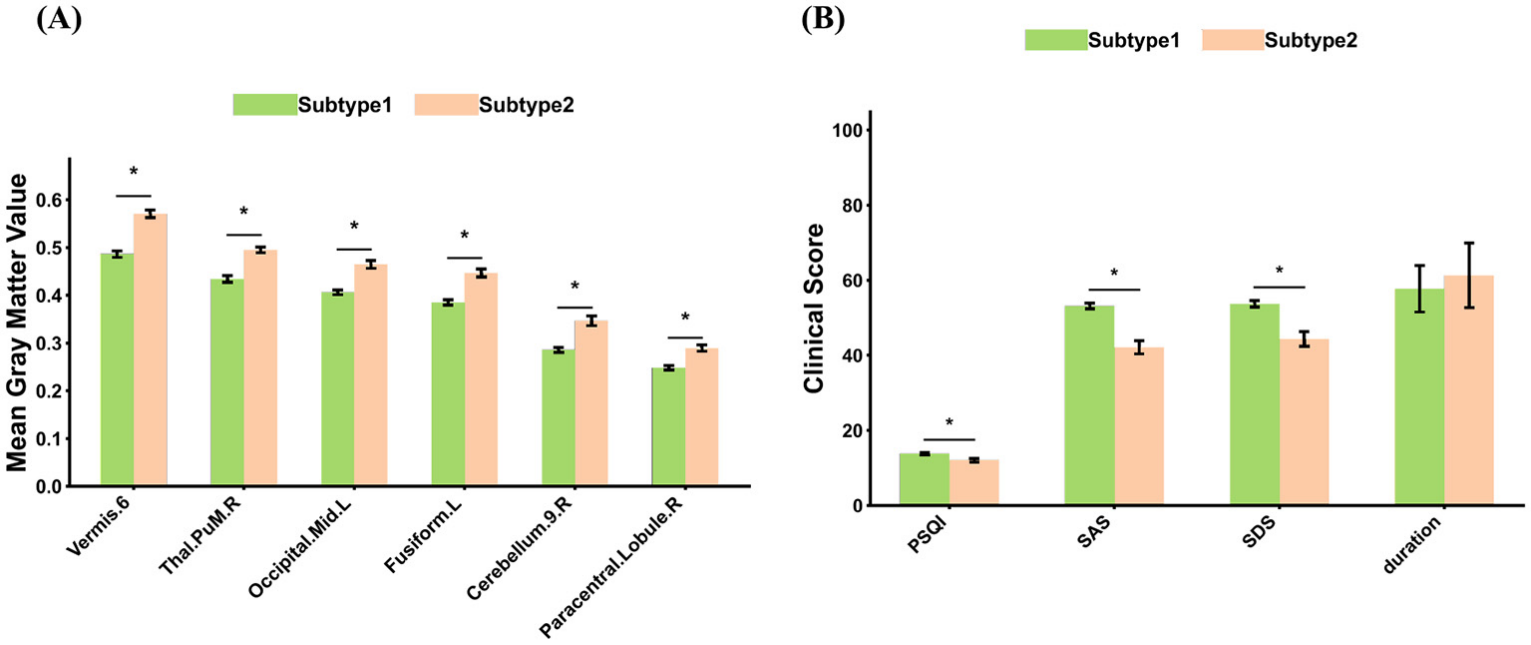
Differences in gray matter volume (GMV) and clinical measures between insomnia disorder subtypes. **(A)** Mean GMV values of brain regions exhibiting significant differences between Subtype 1 and Subtype 2 (*p* < 0.05, FDR corrected). Regions showing significant inter-subtype differences are marked with asterisks. **(B)** Mean scores of clinical measures that showed significant differences between Subtype 1 and Subtype 2 (*p* < 0.05, FDR corrected). Clinical measures with significant inter-subtype differences are marked with asterisks.

As shown in Figure 5A, several brain regions exhibited significant gray matter volume (GMV) differences between the two insomnia subtypes, including the *Vermis.6, Thal.PuM.R, Occipital.Mid.L, Fusiform.L, Cerebellum.9.R*, and *Paracentral.Lobule.R*. Across all these regions, Subtype 1 consistently showed lower mean GMV values compared with Subtype 2, indicating more pronounced gray matter reductions in cerebellar, occipital, and thalamic areas.

Figure 5B summarizes the clinical measures that differed significantly between subtypes. Subtype 1 exhibited significantly higher PSQI, SAS, and SDS scores than Subtype 2 (*p* < 0.05, FDR corrected), reflecting more severe sleep disturbances as well as greater anxiety and depressive symptoms. In contrast, disease duration did not show a significant inter-subtype difference after FDR correction.Overall, these results indicate that Subtype 1 represents a more severe phenotype of insomnia disorder, characterized by both greater gray matter reductions and worse clinical symptomatology compared with Subtype 2.

### 3.4 Associations between GMV and clinical scores in subtype-differentiated regions

**Figure 6:**
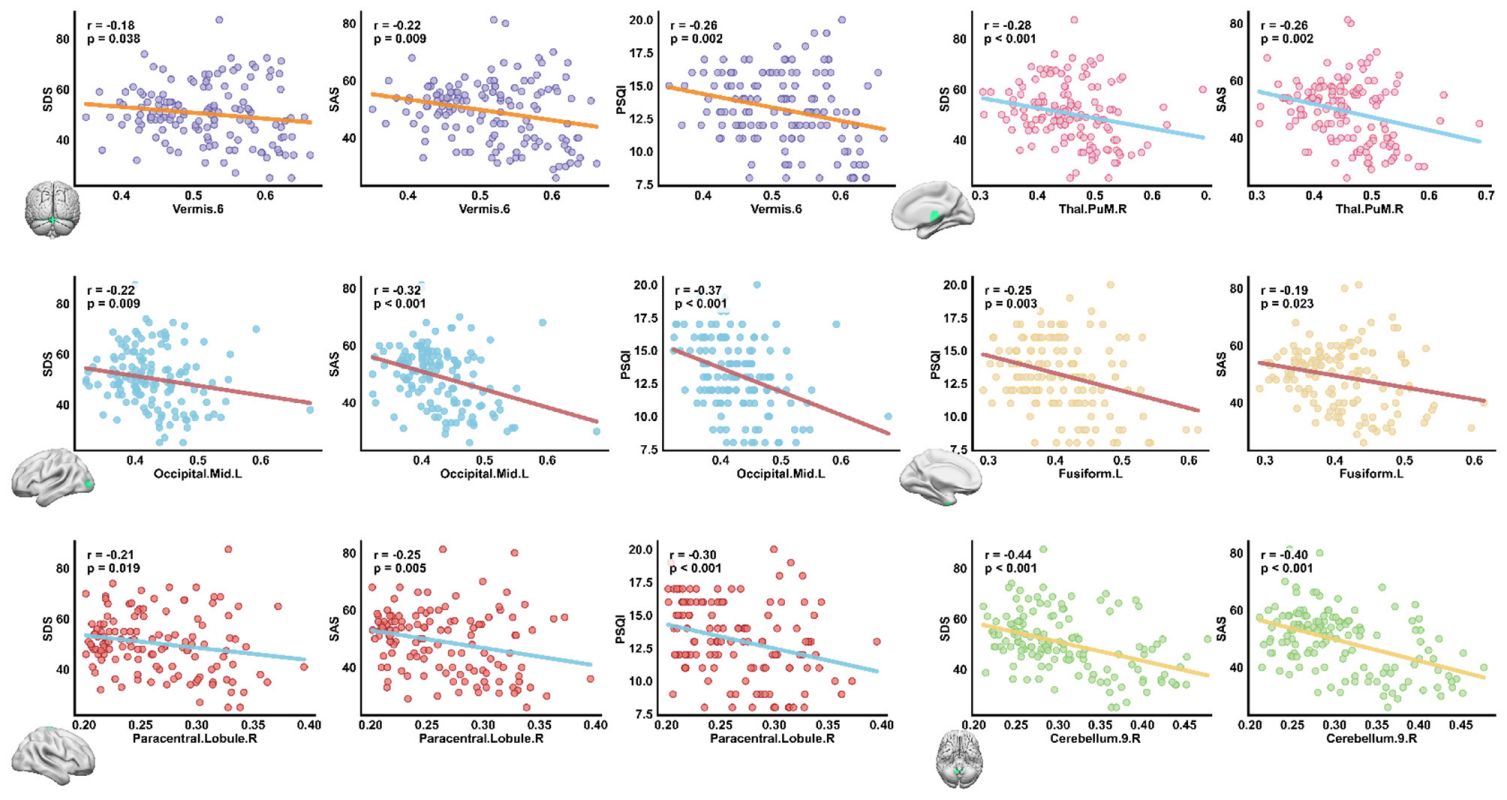
Associations between GMV and clinical scores in subtype-differentiated regions (*p* < 0.05, FDR corrected). Significant negative correlations were observed between GMV and clinical measures (SDS, SAS, PSQI) in several regions: *Vermis.6, Thal.PuM.R, Occipital.Mid.L, Fusiform.L, Paracentral.Lobule.R*, and *Cerebellum.9.R*. Among these, *Cerebellum.9.R* exhibited the strongest correlations with SDS (*r* = −0.44, *p* < 0.001) and SAS (*r* = −0.40, *p* < 0.001).

To further explore the clinical relevance of the structural alterations between subtypes, correlation analyses were conducted between GMV of the subtype-differentiated brain regions and clinical scores, including PSQI, SAS, and SDS. As shown in Figure 6, significant negative correlations were observed across multiple regions, indicating that lower GMV was associated with higher symptom severity.

Specifically, GMV in the *Vermis.6* showed significant negative correlations with SDS (*r* = −0.18, *p* = 0.038), SAS (*r* = −0.22, *p* = 0.009), and PSQI (*r* = −0.26, *p* = 0.002). Similarly, the *Thal.PuM.R* exhibited negative correlations with SDS (*r* = −0.28, *p* < 0.001) and SAS (*r* = −0.26, *p* = 0.002). The *Occipital.Mid.L* showed robust associations with SDS (*r* = −0.22, *p* = 0.009), SAS (*r* = −0.32, *p* < 0.001), and PSQI (*r* = −0.37, *p* < 0.001). Negative correlations were also observed in the *Fusiform.L* with PSQI (*r* = −0.25, *p* = 0.003) and SAS (*r* = −0.19, *p* = 0.023), and in the *Paracentral.Lobule.R* with SDS (*r* = −0.21, *p* = 0.019), SAS (*r* = −0.25, *p* = 0.005), and PSQI (*r* = −0.30, *p* < 0.001). Finally, the *Cerebellum.9.R* demonstrated the strongest negative associations, with SDS (*r* = −0.44, *p* < 0.001) and SAS (*r* = −0.40, *p* < 0.001).

Collectively, these findings indicate that reductions in GMV within cerebellar, occipital, and thalamic regions were significantly associated with increased insomnia severity, anxiety, and depressive symptoms, suggesting that these regions play critical roles in the neuropathophysiological mechanisms underlying insomnia disorder.

### 3.5 IDSCN differences between insomnia subtypes

**Figure 7:**
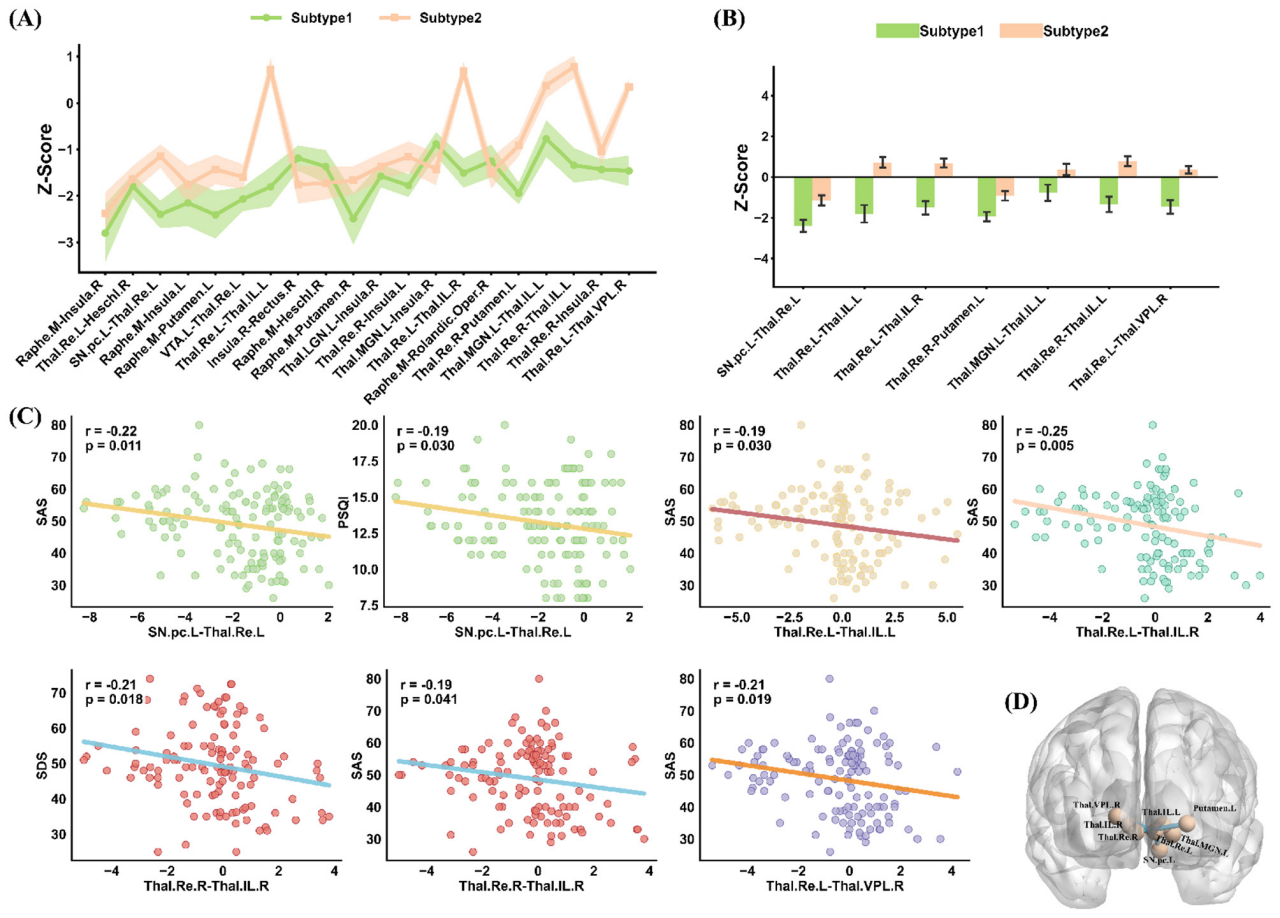
Structural covariance edge analysis across ID subtypes. **(A)** Z-scores of the top 20 structural covariance edges showing the greatest number of significant alterations between the two ID subtypes. **(B)** The seven structural edges exhibiting significant differences between Subtype 1 and Subtype 2 (*p* < 0.05). **(C)** Correlations between altered structural covariance edges and clinical scores, showing only statistically significant associations after FDR correction (*p* < 0.05). **(D)** Spatial visualization of the seven differential edges in the brain.

The Z-scores of the top 20 structural covariance edges in the two ID subtypes are presented in Figure 7A. Among these top 20 edges, seven showed significant differences in their Z-scores (*p* < 0.05) between the two ID subgroups, as illustrated in Figure 7B. Specifically, Subtype 1 exhibited lower Z-scores than Subtype 2 in the following connections: *SN.pc.L-Thal.Re.L, Thal.Re.L-Thal.IL.L, Thal.Re.L-Thal.IL.R, Thal.Re.R-Putamen.L, Thal.MGN.L-Thal.IL.L, Thal.Re.R-Thal.IL.L*, and *Thal.Re.L-Thal.VPL.R*.

As shown in Figure 7C, among the top 20 edges, *SN.pc.L-Thal.Re.L, Thal.Re.L-Thal.IL.L, Thal.Re.L-Thal.IL.R, Thal.Re.L-Thal.VPL.R*, and *Thal.Re.R-Thal.IL.R* were significantly negatively correlated with clinical scores. Figure 7D visualizes the spatial locations of these seven differential edges in the brain. Using Z-scores of the top 20 IDSCN edges as features, we obtained an alternative two-cluster solution. As shown in Supplementary Figure S3.A, correspondence with GM-PGAAE-derived subtypes was limited: 70 of 90 GM-PGAAE Subtype 1 subjects overlapped with IDSCN Subtype 1, whereas only 10 of 50 GM-PGAAE Subtype 2 subjects overlapped with IDSCN Subtype 2. This asymmetric overlap suggests that the two approaches capture different aspects of inter-individual heterogeneity. Notably, IDSCN-based subtypes showed no significant differences in PSQI, SAS, SDS, or illness duration (Supplementary Figure S3.B), unlike the GM-PGAAE-derived subtypes.

## 4 Discussion

Graph-based learning frameworks have recently emerged as a powerful means to jointly model clinical features and neuroimaging data. By representing each individual as a node and defining edges based on intersubject similarity, population-graph models are able to capture complex regional and network-level relationships. Prior work—including TGML (Li et al., 2025), aggregator-normalization GCNs (Salim and Hamza, 2024), and graph attention-based models such as GAT-AE and MSTGAT (Velickovic et al., 2017; Salehi and Davulcu, 2019; Liu et al., 2025)—has demonstrated strong performance in brain disorder classification, highlighting the ability of graph architectures to extract meaningful representations from multimodal data. However, these approaches have focused primarily on diagnostic prediction, with relatively little emphasis on characterizing disorder heterogeneity or deriving clinically interpretable subtypes. Moreover, most existing models rely on functional or multimodal inputs, leaving the integration of structural MRI with clinical symptoms in a unified graph framework comparatively underexplored.

Within this context, our study extends graph-based neuroimaging research by applying a population-graph representation learning model to characterize heterogeneity in insomnia disorder. The proposed GM-PGAAE generated low-dimensional embeddings that captured informative intersubject relationships, and clustering of these embeddings revealed distinct subgroups. These findings suggest that population-graph approaches can uncover latent clinical and neurobiological variability that might not be detected by traditional clinical or imaging-only analyses.

To further contextualize the proposed framework, we compared GM-PGAAE with several baseline approaches, including alternative graph neural network autoencoders (GCN-AE and GraphSAGE-AE) as well as non-graph clustering methods based on ROI-level gray matter volume features. While these baseline methods were able to produce stable clustering solutions and, in some cases, detect clinical differences between subgroups, they generally exhibited lower cluster separation and less consistent clinical differentiation across symptom dimensions. In comparison, GM-PGAAE achieved higher silhouette coefficients and robust clustering stability, while simultaneously yielding subtypes that were differentiated across multiple core clinical symptom scales. The attention-based aggregation mechanism in GM-PGAAE may provide a more flexible means of modeling heterogeneous intersubject relationships in population graphs, which is particularly advantageous for unsupervised subtype discovery where no explicit labels are available.

Two distinct subtypes of insomnia disorder were identified using the proposed GM-PGAAE framework. Compared with Subtype 2, Subtype 1 exhibited significantly higher clinical scores across all symptom dimensions, including sleep disturbance (PSQI), anxiety (SAS), and depression (SDS). Correspondingly, Subtype 1 showed reduced gray matter volumes in several brain regions, including the *Vermis.6, Thal.PuM.R, Occipital.Mid.L, Fusiform.L, Cerebellum.9.R*, and *Paracentral.Lobule.R*. Correlation analyses further revealed that the GMV of these regions was negatively associated with clinical symptom severity, suggesting that greater structural loss in these areas is linked to more pronounced insomnia-related symptoms. Moreover, IDSCN analysis demonstrated that Subtype 1 exhibited significantly lower Z-scores than Subtype 2 in several thalamocortical and subcortical connections, Together, these findings delineate two biologically and clinically distinct subtypes of insomnia, characterized by differing patterns of gray matter alterations and network-level structural connectivity.

The voxel-wise analyses revealed robust gray matter alterations distinguishing the two insomnia subtypes and further differentiating each subtype from healthy controls. Subtype 1 showed pronounced gray matter reductions relative to Subtype 2 in the *Vermis 6, Thal.PuM.R, Occipital.Mid.L, Fusiform.L, Cerebellum 9*, and *Paracentral.Lobule.R*. These regions are tightly involved in arousal regulation, emotional processing, and sensory-motor integration. For example, the cerebellar vermis plays a critical role in emotion and autonomic regulation, and structural alterations in this region have been reported in insomnia and other hyperarousal-related disorders (Lin et al., 2023; Ma et al., 2023). Thalamic abnormalities–particularly within the pulvinar–have also been linked to impaired thalamocortical gating and heightened sensory reactivity in chronic insomnia (Shao et al., 2025). Similarly, reductions in the occipital and fusiform cortices have been observed in patients with chronic insomnia and are thought to reflect disturbances in visual-emotional integration and nocturnal hypervigilance (Wang et al., 2025; Zou et al., 2021).

Importantly, these structural differences co-occurred with substantially worse clinical symptomatology in Subtype 1, including higher PSQI, SAS, and SDS scores. The robust negative correlations observed between GMV in the subtype-differentiated regions and clinical severity further support the functional relevance of these structural alterations. For instance, the cerebellar vermis and posterior cerebellar lobules showed strong associations with anxiety and depressive symptoms, consistent with their established roles in affect regulation and autonomic control. Similarly, the significant correlations between pulvinar GMV and symptom scores reinforce the involvement of thalamic nuclei in hyperarousal and emotion-sensory integration disturbances characteristic of insomnia. The occipital and fusiform regions also demonstrated substantial negative correlations with PSQI, SAS, and SDS. Given the occipital cortex's involvement in visual salience and the fusiform gyrus's role in affective perceptual processing, reduced GMV in these regions may contribute to excessive sensory responsiveness and attentional bias toward sleep-related threats, mechanisms commonly implicated in chronic insomnia.

Compared with healthy controls, Subtype 1 also exhibited reduced gray matter in the insula, putamen, superior temporal gyrus, and lingual gyrus. These findings are consistent with previous studies demonstrating insular dysfunction in salience processing and interoceptive regulation in insomnia (Reimann et al., 2025), as well as putamen atrophy linked to impaired sensorimotor and habitual processing in chronic insomnia disorder (Emamian et al., 2021). Gray matter reductions in temporal and lingual cortices have likewise been reported in insomnia cohorts and associated with altered auditory processing and visual-emotional integration (Falgàs et al., 2021). In contrast, Subtype 2 demonstrated extensive gray matter increases relative to healthy controls, particularly in the occipital cortex (calcarine, lingual, cuneus), cerebellar vermis, supplementary motor area, and thalamic subregions. Prior sleep research has interpreted occipital GMV enlargement as a compensatory mechanism reflecting heightened visual network engagement or neuroplastic adaptation after sleep disturbance (Grau-Rivera et al., 2020). Increases in cerebellar and thalamic gray matter similarly align with studies reporting neuroadaptive changes in individuals with subthreshold insomnia or relatively preserved sleep-wake regulation. The enlarged supplementary motor area and rectus regions further correspond to findings that milder or compensated insomnia may involve preserved or increased fronto-striatal structural integrity (Huang et al., 2024).

The individualized differential structural covariance network (IDSCN) analysis revealed that Subtype 1 exhibited significantly reduced Z-scores across a set of thalamocortical and subcortical connections, including: *SN.pc.L-Thal.Re.L, Thal.Re.L-Thal.IL.L/R, Thal.Re.R-Putamen.L, Thal.MGN.L-Thal.IL.L, Thal.Re.R-Thal.IL.L*, and *Thal.Re.L-Thal.VPL.R*. Importantly, this subtype-specific pattern aligns with emerging evidence showing that insomnia is not a unitary disorder but comprises distinct neurobiological subtypes with dissociable thalamo-cortical and thalamo-striatal alterations. Recent large-scale structural connectivity work demonstrated that different insomnia subtypes exhibit unique deviation profiles across thalamic, striatal, and cortico-limbic circuits rather than sharing a homogeneous deficit pattern (Bresser et al., 2025). The convergence between those subtype-specific abnormalities and the reduced thalamic relay and intralaminar covariance observed in Subtype 1 underscores thalamic and basal ganglia pathways as a major axis of neurobiological differentiation in insomnia. Moreover, our findings resonate with recent multivariate analyses showing that sleep health domains co-vary with functional connectivity patterns centered on attentional and thalamic networks, forming a distributed connectome phenotype with diagnostic relevance for insomnia (Wang et al., 2023). This supports the view that thalamic network integrity reflects a broader multi-dimensional sleep-health construct, consistent with the widespread covariance disruptions observed in Subtype 1. Finally, whole-brain diffusion imaging studies further demonstrate that reduced fronto-subcortical structural connectivity–particularly involving the left insula and its projections to frontal and subcortical targets–is a core feature of Insomnia Disorder (Jespersen et al., 2020). This insula-centered disconnection pattern is closely mirrored in our data, where Subtype 1 showed marked reductions in thalamic and thalamo-striatal covariance. Given the insula's essential role in interoception, arousal regulation, and slow-wave sleep generation, the combined evidence suggests that Subtype 1 reflects a structurally vulnerable phenotype characterized by broad disruptions across a unified fronto-insula-thalamic regulatory axis. Such network-level impairment may underlie the heightened clinical severity observed in this subtype. To further contextualize the proposed framework, we additionally explored an alternative clustering strategy based on Z-scores of IDSCN edges. This network-based clustering showed limited correspondence with the GM-PGAAE-derived subtypes and did not yield significant clinical differentiation. These findings suggest that edge-level covariance perturbations and population-level representation learning capture complementary aspects of inter-individual heterogeneity, underscoring the value of integrative graph-based models for subtype discovery.

Although the present study provides a structurally informed framework for identifying insomnia subtypes, several methodological limitations should be noted. Specifically, our analysis relied solely on gray-matter morphology and did not incorporate resting-state functional connectivity, which has been shown in previous studies to capture complementary network-level alterations across various psychiatric conditions (e.g., Zhang et al., 2025; Sun et al., 2024). Integrating functional connectivity in future work could further enhance the identification and characterization of insomnia subtypes. In addition, the IDSCN analysis relied on a healthy control group as the reference for Z-score computation. The relatively modest size of the healthy control sample may influence the stability of individual-level Z-scores and introduce sensitivity to group-level variability. Accordingly, IDSCN-related findings are intended to complement the primary subtype analyses rather than serve as standalone statistical evidence. Future studies with larger reference cohorts or independent datasets will be important to further evaluate the robustness of the observed network-level alterations. Finally, cross-dataset validation of the identified subtypes was not performed. While external validation is strongly encouraged in clustering and subtype discovery studies to assess generalizability, suitable independent datasets with comparable imaging protocols, clinical measures, and diagnostic criteria for insomnia disorder were not available. Future studies leveraging larger multi-site cohorts or harmonized datasets will be essential to further evaluate the robustness and generalizability of the present findings.

Collectively, these findings indicate that insomnia disorder encompasses distinct neurobiological subtypes, characterized by divergent patterns of gray matter structure and covariance. Subtype 1 represents a high-burden phenotype, marked by widespread GMV reductions across arousal, sensory, and affective regulatory circuits, accompanied by markedly elevated clinical symptoms and pervasive covariance breakdown within thalamo-striatal and thalamo-sensory networks. This strong structure-symptom coupling suggests a more severe and biologically vulnerable subtype. In contrast, Subtype 2 exhibits relatively preserved–or even enhanced–gray matter and structural coordination in occipital-cerebellar-thalamic networks, consistent with a potentially neurocompensatory or resilience-related profile. These contrasting structural and covariance patterns underscore the heterogeneity of insomnia and highlight the potential clinical utility of GMV-based and thalamic-centered covariance markers for stratifying patients, identifying those at elevated risk for more severe manifestations, and informing individualized treatment strategies.

## Data Availability

The raw data supporting the conclusions of this article will be made available by the authors, without undue reservation.
