# Supplementary material for: Subtyping insomnia disorder with a population graph attention autoencoder: revealing two distinct biotypes
Source: Front Neurosci. 2026 Feb 11;20:1766155. doi: 10.3389/fnins.2026.1766155 (PMC12932581; doi:10.3389/fnins.2026.1766155)
Supplement: Supplementary file 1 [file Data_Sheet_1.docx]

**Supplementary Materials for**

[Subtyping Insomnia Disorder with a Population Graph Attention Autoencoder: Revealing Two Distinct Biotypes](https://review.frontiersin.org/Document/DownloadPDF?articleId=1766155&userId=3308672&roleId=16)

**Supplementary Tables**

**Table S1.** Regions with significant differences identified in all ID Subtype1 vs. Subtype2.

| **Brain regions** | **MNI Coordinates** | | | ***t* values** | **Cluster size  (voxels)** |
| --- | --- | --- | --- | --- | --- |
|  | **x** | **y** | **z** |  |  |
| **Subtype1<Subtype2** |  |  |  |  |  |
| Vermis.6 | 0 | -70.5 | -15 | -8.64046 | 16392.4 |
| Thal.PuM.R, | -3 | -24 | 3 | -7.37493 | 2197.12 |
| Occipital.Mid.L | -37.5 | -91.5 | 3 | -7.67711 | 516.375 |
| Fusiform.L | -24 | -1.5 | -51 | -6.35538 | 462.375 |
| Cerebellum.9.R, | 0 | -49.5 | -63 | -24.5654 | 300.375 |
| Paracentral_Lobule_R | 12 | -25.5 | 78 | -6.12036 | 178.875 |

**Table S2.** Regions with significant differences identified in two subtypes ID vs.HC.

|  | **Brain regions** | **MNI Coordinates** | | | ***t* values** | **Cluster size  (voxels)** |
| --- | --- | --- | --- | --- | --- | --- |
|  |  | **x** | **y** | **z** |  |  |
| **Subtype 1** | **ID<HC** |  |  |  |  |  |
|  | Insula.L | -31.5 | 9 | 1.5 | -10.2613 | 8930.25 |
|  | Cerebellum.4.5.L | -10.5 | -51 | -10.5 | -7.64061 | 2686.5 |
|  | Putamen.R | 25.5 | 15 | 7.5 | -7.24778 | 1711.12 |
|  | Heschl.R | 45 | -24 | 9 | -7.6389 | 1609.88 |
|  | Temporal.Inf.L | -25.5 | -10.5 | -45 | -7.1305 | 1221.75 |
|  | Temporal.Mid.L | -48 | -16.5 | -12 | -6.80155 | 799.875 |
|  | Temporal.Mid.L | -46.5 | -42 | 6 | -6.52274 | 688.5 |
|  | Precentral.R | 19.5 | -19.5 | 76.5 | -6.74007 | 580.5 |
|  | Temporal.Pole.Sup.R | 36 | 4.5 | -19.5 | -8.40251 | 563.625 |
|  | **ID>HC** |  |  |  |  |  |
|  | Cerebellum.8.L | -21 | -45 | -57 | 8.2211 | 3628.12 |
|  | N.Acc.L | -7.5 | -1.5 | -9 | 9.64797 | 2446.88 |
|  | Calcarine.L | -1.5 | -97.5 | 7.5 | 6.99946 | 1890 |
|  | Supp.Motor.Area.L | -4.5 | -16.5 | 61.5 | 7.35868 | 769.5 |
| **Subtype 2** | **ID>HC** |  |  |  |  |  |
|  | Calcarine.L | 0 | -91.5 | -4.5 | 10.8565 | 5923.12 |
|  | Vermis.6 | 1.5 | -57 | -27 | 9.17127 | 3034.12 |
|  | Cerebellum.8.L | -21 | -43.5 | -58.5 | 7.94622 | 2797.88 |
|  | Thal.LGN.L | -19.5 | -31.5 | -3 | 9.00262 | 1559.25 |
|  | Thal.VA.R | -6 | -3 | -6 | 8.61068 | 1414.12 |
|  | Cerebellum.9.R | 0 | -49.5 | -64.5 | 26.5302 | 1059.75 |
|  | Rectus.R | 4.5 | 22.5 | -27 | 8.1102 | 799.875 |
|  | Supp.Motor.Area.L | -3 | -15 | 60 | 6.60132 | 580.5 |
|  | Fusiform.L | -46.5 | -64.5 | -21 | 6.81725 | 432 |
|  | Cerebellum.8.R | 13.5 | -67.5 | -61.5 | 6.44441 | 199.125 |
|  | Occipital.Mid.L | -33 | -96 | 3 | 6.58997 | 195.75 |
|  | **ID<HC** |  |  |  |  |  |
|  | Insula.L | -33 | 7.5 | 0 | -6.99554 | 1032.75 |

**Supplementary Figures**

**
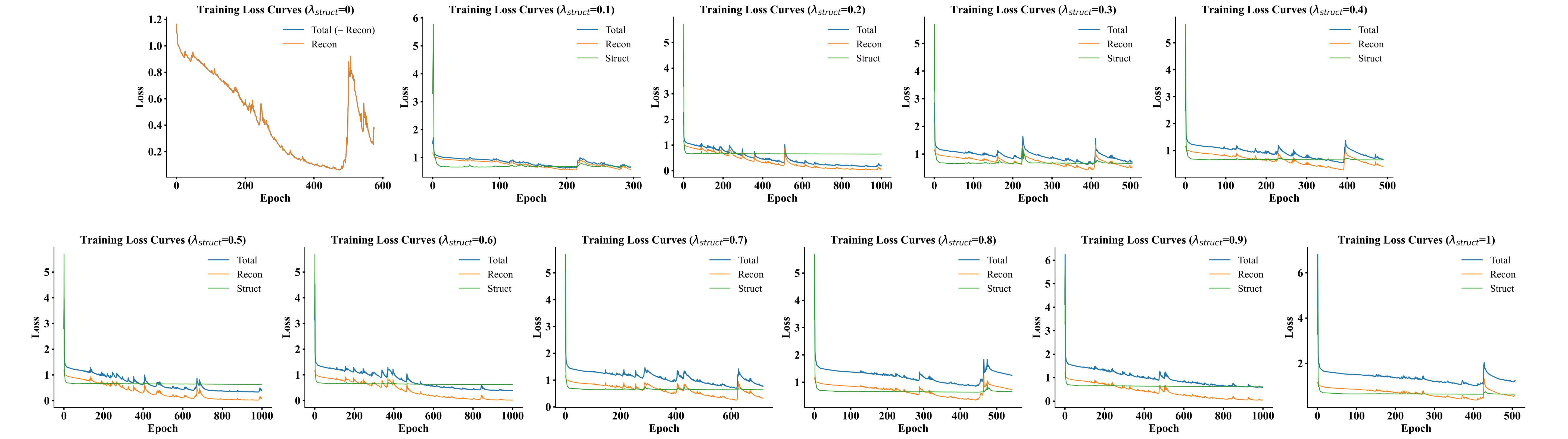
**

**Figure S1 .**Training loss curves of the GM-PGAAE model under different values of the structural loss weight λ. For each λ setting, the total loss, reconstruction loss, and structural loss are shown across training epochs.


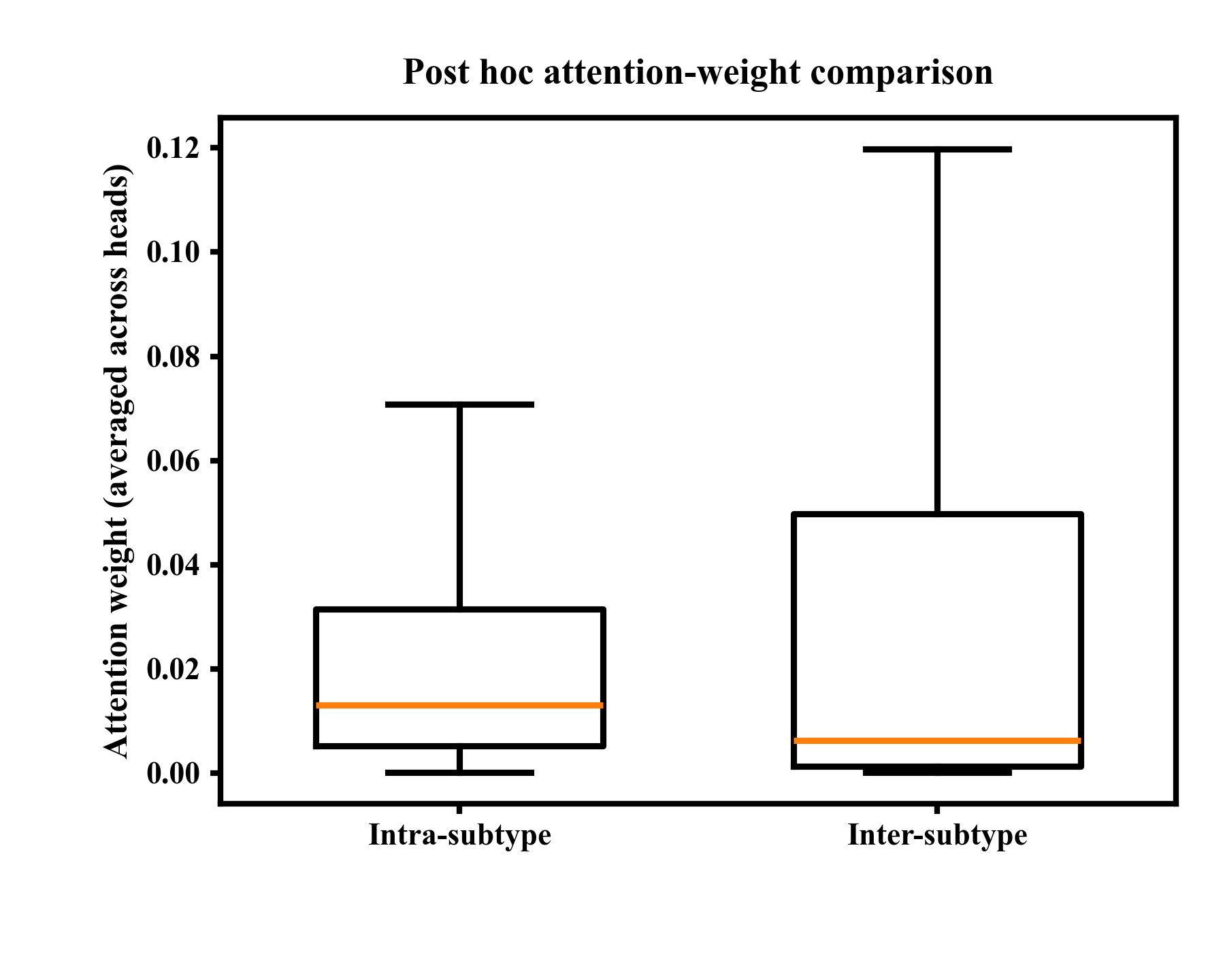


**Figure S2.** Distribution of attention weights for intra-subtype and inter-subtype edges. Attention weights were extracted from the GAT encoder after training and grouped according to the final K-means subtype labels. The inter-subtype edges showed higher mean attention weights than intra-subtype edges, and the difference was assessed using a permutation test (p = 2.0 × 10^-^⁴).

**
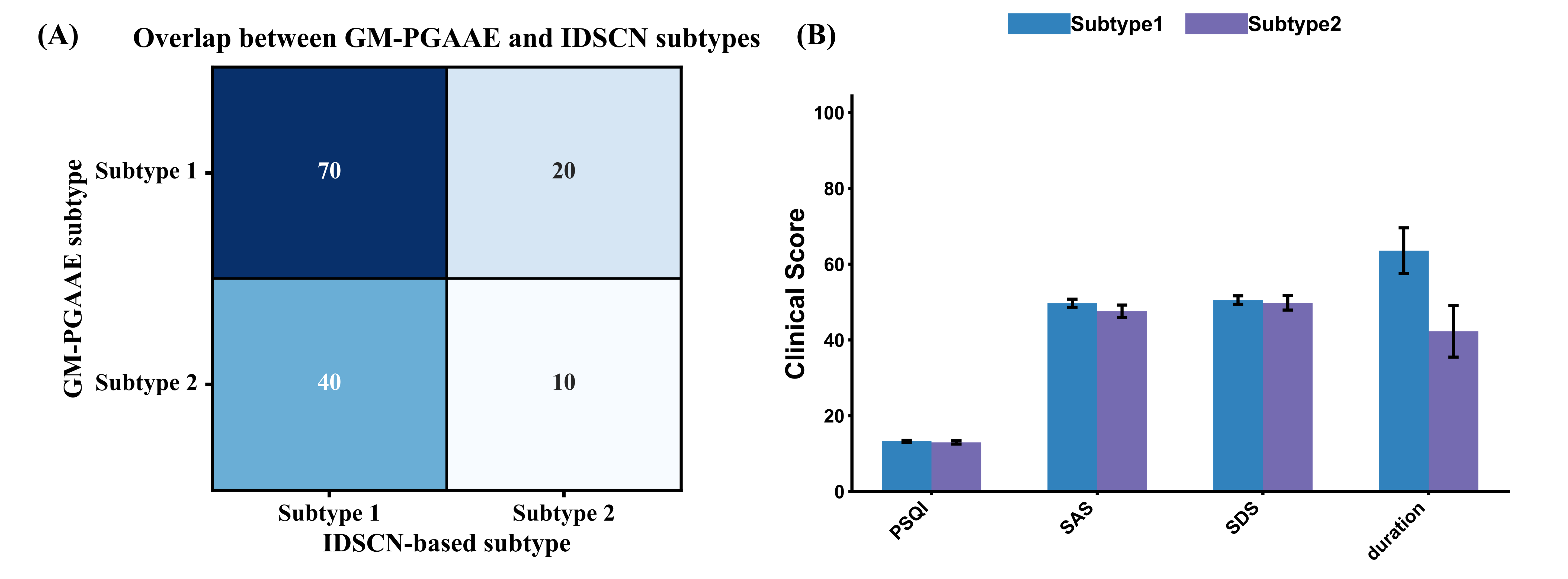
**

**Figure S3.**Comparison between GM-PGAAE derived subtypes and IDSCN-based clustering results. (A) Overlap between subtypes identified by the proposed GM-PGAAE framework and those obtained using k-means clustering on IDSCN edge-based features. Cell values indicate the number of subjects assigned to each subtype combination, illustrating a partial correspondence between the two clustering approaches. (B) Clinical score comparisons (PSQI, SAS, SDS, and illness duration) across subtypes defined by IDSCN-based clustering. No significant differences were observed between the two IDSCN-derived subtypes across these clinical measures, in contrast to the GM-PGAAE derived subtypes reported in the main analysis.
